# Using clotted, pelleted blood samples for direct molecular detection of *Bartonella* spp. in small mammal wildlife surveillance studies

**DOI:** 10.1186/s13104-024-06841-5

**Published:** 2024-07-02

**Authors:** Simon P. Jeeves, Champika Fernando, Jonathon D. Kotwa, Samira Mubareka, Janet E. Hill, Claire M. Jardine

**Affiliations:** 1 Department of Pathobiology, University of Guelph, 50 Stone Rd E, Guelph, ON N1G 2W1 Canada; 2 Department of Veterinary Microbiology, University of Saskatchewan, 52 Campus Dr, Saskatoon, Saskatchewan, S7N 5B4 Canada; 3 Sunnybrook Research Institute, 2075 Bayview Ave, Toronto, ON M4N 3M5 Canada; 4 Department of Laboratory Medicine and Pathobiology, Faculty of Medicine, University of Toronto, 1 King’s College Circle, Toronto, ON M5S 1A8 Canada; 5 Ontario-Nunavut, Department of Pathobiology, Canadian Wildlife Health Cooperative, University of Guelph, 50 Stone Rd E, Guelph, ON N1G 2W1 Canada

**Keywords:** *Bartonella*, Blood clot, DNA extraction, Small mammal, Surveillance, PCR

## Abstract

**Objective:**

*Bartonella* are emerging bacterial zoonotic pathogens. Utilization of clotted blood samples for surveillance of these bacteria in wildlife has begun to supersede the use of tissues; however, the efficacy of these samples has not been fully investigated. Our objective was to compare the efficacy of spleen and blood samples for DNA extraction and direct detection of *Bartonella* spp. via qPCR. In addition, we present a protocol for improved DNA extraction from clotted, pelleted (i.e., centrifuged) blood samples obtained from wild small mammals.

**Results:**

DNA concentrations from kit-extracted blood clot samples were low and A260/A280 absorbance ratios indicated high impurity. Kit-based DNA extraction of spleen samples was efficient and produced ample DNA concentrations of good quality. We developed an in-house extraction method for the blood clots which resulted in apposite DNA quality when compared to spleen samples extracted via MagMAX DNA Ultra 2.0 kit. We detected *Bartonella* in 9/30 (30.0%) kit-extracted spleen DNA samples and 11/30 (36.7%) in-house-extracted blood clot samples using PCR. Our results suggest that kit-based methods may be less suitable for DNA extraction from blood clots, and that blood clot samples may be superior to tissues for *Bartonella* detection.

**Supplementary Information:**

The online version contains supplementary material available at 10.1186/s13104-024-06841-5.

## Introduction

The genus *Bartonella* is a group of Gram-negative, fastidious, facultative intraerythrocytic bacteria in the family Bartonellaceae [1]. Species of *Bartonella* are regarded as emerging zoonotic pathogens, transmitted from vertebrate animal hosts to humans via arthropod vectors such as fleas, biting flies, and lice [1]. Twenty-five recognized species of *Bartonella* have been isolated from wild small mammal hosts such as rodents and shrews, highlighting the involvement of these animals in sylvatic *Bartonella* cycles [1–3]. There is an incomplete understanding of *Bartonella* reservoir ecology, and novel *Bartonella* species continue to be detected in different wildlife hosts and new geographic areas, emphasizing the need for continued surveillance [1, 4].

Despite the abundance of molecular techniques (e.g., PCR) available for testing biological samples for presence of bacterial pathogens, the majority of *Bartonella* surveillance research continues to employ methodologies which rely on successful culture and isolation [3]. This typically employs use of blood and – particularly in the case of small mammals – homogenized tissues to culture *Bartonella* isolates on solid media [5, 6]. In many wildlife disease surveillance settings, sample sizes and resources are limited which can make laborious culture-based methodologies challenging. Furthermore, appropriate tissue samples are not always available; spleen, for example, is collected post-mortem and cannot be collected in studies where only live animals are sampled. Blood samples however, can be obtained from live animals and can be used for multiple testing modalities – especially when centrifugated and fractionated into their different components. Our objective was to compare the efficacy of spleen and blood samples for DNA extraction and direct detection of *Bartonella* spp. via qPCR. In addition, we present a protocol for improved DNA extraction from clotted, pelleted (i.e., centrifuged) blood samples obtained from wild small mammals.

## Methods

Samples for this study were taken from animals previously collected for an ongoing surveillance project investigating zoonotic pathogen occurrence in urban small mammal wildlife in Toronto, Canada, in 2021 and 2022. Trapping and sampling procedures have been previously described [7]. Briefly, small mammal species were live-trapped using Sherman traps (H.B. Sherman Traps Inc., Tallahassee, FL, USA) baited with sunflower seeds and apple. Identification of animals was made on a morphological basis by trained field personnel. Animals were humanely euthanized promptly using open-drop inhaled isoflurane in an enclosed chamber. Euthanasia was followed by cardiac puncture to obtain 0.5 mL whole blood. Samples were kept on ice until processing later the same day. Whole blood samples were centrifuged at 3000 × *g* for 10 min followed by separation of sera from clotted blood. Tissue samples were obtained at necropsy on the same day. All samples were stored at -80 °C until further use.

For the purposes of this study, we tested thirty animals with both blood and spleen samples. This included 27 white-footed mice (*Peromyscus leucopus*), two eastern meadow voles (*Microtus pennsylvanicus*), and one short-tailed shrew (*Blarina brevicauda*).

For DNA extraction and PCR, samples were shipped on dry ice to the Department of Veterinary Microbiology, University of Saskatchewan (Saskatoon, Canada). The MagMAX DNA Ultra 2.0 kit (Thermo Fisher Scientific Inc., Canada) was used as a baseline extraction procedure to compare efficacy of a commercial kit method against the in-house protocol described herein; 60–120 mg of blood clot (BC) was extracted according to the manufacturer’s instructions. To compare the efficacy of BC DNA extraction to that of tissues, 60 mg of spleen samples from the same animals were extracted using the aforementioned kit following the manufacturer’s instructions. The in-house procedure for DNA extraction from BC samples was a salting-out technique adapted from Martín-Platero et al. [8]. Briefly, 50–100 mg of BC was added to 500 µL of MagMAX Cell and Tissue DNA Extraction Buffer (Thermo Fisher Scientific Inc., Canada) in a 2 mL Lysing Matrix E tube (MP Biomedicals LLC, USA). Samples were homogenized in a bead beater for 2 min at 30 beats/sec. Samples were then vortexed for 5 min. Next, 20 µL of MagMAX DNA Multi-Sample Ultra 2.0 Enhancer Solution (Thermo Fisher Scientific Inc., Canada) and 40 µL of MagMAX DNA Multi-Sample Ultra 2.0 Proteinase K solution (100 mg/mL; Thermo Fisher Scientific Inc., Canada) were added to the samples, followed by water bath incubation at 55 °C for 2 h with brief vortexing every 30 min. Samples were then centrifuged at 3500 × *g* for 1 min and supernatants were transferred to clean 1.5 mL microcentrifuge tubes. Next, 200 µL of sodium acetate (3.0 M, pH 5.2) was added, followed by inverting to mix and incubation on ice for 15 min. Samples were then centrifuged at 16,200 × *g* for 10 min. Then, 400 µL of supernatant was added to 400 µL of 100% isopropanol, followed by addition of 1 µL of UltraPure Glycogen (Thermo Fisher Scientific Inc., Canada). Samples were then mixed in a tube rotator for 5 min at room temperature, followed by centrifugation at 16,200 × *g* for 5 min. Supernatant was discarded and pellet was allowed to dry before adding 400 µL of fresh 70% ethanol and inverting to mix. Samples were centrifuged at 16,200 × *g* for 3 min. Supernatant was discarded and the pellet was allowed to dry for 5–10 min. DNA was eluted through addition of 100 µL of 1× TE buffer (pH 8.0) and water bath incubation at 65 °C for 1 h. DNA extracts were stored at -20 °C until further analysis. All extraction procedures included controls with reagents only. All samples were diluted 1:10 in molecular-grade water to dilute PCR inhibitors. Quantification of extracted DNA was completed using a NanoDrop ND2000 spectrophotometer (Thermo Fisher Scientific Inc., Canada).

To assess extract suitability for *Bartonella* spp. detection, a SYBR Green real-time PCR (qPCR) was performed to amplify a fragment of the host cytochrome B gene (*cytB*). Primers and product sizes for host *cytB* qPCR are available in Supplementary Table 1. Each *cytB* qPCR reaction was 10 µL in total volume, consisting of: 5 µL of 2× SYBR Green mix (Bio-Rad Laboratories, Inc., USA), 0.5 µM of each primer, 2 µL of molecular-grade water, and 2 µL of template DNA. Extraction controls and no template controls (NTCs) were included in each qPCR batch. The *cytB* qPCR was performed on a Bio-Rad CFX96 (Bio-Rad Laboratories, Inc., USA) with the following thermocycling conditions: 95 °C for 3 min followed by 40 cycles of 95 °C for 10 s, 57 °C for 10 s, 72 °C for 30 s; a melt curve analysis was performed following thermocycling. Samples positive for host *cytB* were selected to advance to *Bartonella* spp. qPCR; negative samples were further diluted to 1:100 in molecular-grade water and re-tested.

Testing for presence of *Bartonella* spp. was done with a SYBR Green qPCR targeting a ~ 380 bp fragment of the *Bartonella* citrate synthase gene (*gltA*) using primers BhCS.781p (5’- GGGGACCAGCTCATGGTGG − 3’) and BhCS.1137n (5’- AATGCAAAAAGAACAGTAAACA − 3’) [9]. Each *gltA* qPCR reaction was 10 µL in total volume, with the same proportions of reagents as the *cytB* qPCR. The positive control, previously described in Himsworth et al. (2020), was a PCR product of *Bartonella vinsonii gltA* prepared at a concentration of 1.03 × 10^6^ copies/µL [10]. Extraction controls and NTCs were included in each qPCR batch. All samples and controls were run in duplicate. The *gltA* qPCR was performed on a Bio-Rad CFX96 (Bio-Rad Laboratories, Inc., USA) with the same thermocycling conditions as the *cytB* qPCR; a melt curve analysis was performed following thermocycling. Samples with a Ct value < 40 and melt peak at 80.0° C to 81.0° C were considered positive for *Bartonella* spp. A subset of positive samples from both BCs and spleen samples were sent for Sanger sequencing to confirm amplification of *Bartonella gltA*.

## Results

Overall, BCs extracted via the in-house method were comparable in quality to those obtained from spleen samples extracted via kit method (Table 1). DNA concentrations of spleen sample kit extracts (median 444.1 ng/µL, mean 458.1 ng/µL) were substantially higher than that of BC in-house extracts (median 33.2 ng/µL, mean 49.3 ng/µL), likely due to the high cellular content of tissue samples. Notably, BC samples extracted via kit were determined to be unsuitable for further analysis; DNA concentrations were mostly low or unmeasurable (median 3.1 ng/µL, mean 11.7 ng/µL) and A260/A280 measurements suggested presence of impurities (Table 1).

**Table 1:** Concentrations and purities of DNA extractions from spleen and blood clot samples

	MagMAX™ DNA Ultra 2.0 kit				In-house method	
Sample ID	Spleen		Blood clot		Blood clot	
	[DNA] (ng/µL)	260/280	[DNA] (ng/µL)	260/280	[DNA] (ng/µL)	260/280
21-SM-02	1114.2	1.86	-2.9	3.5	N/A ^a^	N/A
21-SM-03	440.2	1.87	-2.8	3.29	N/A	N/A
21-SM-04	-3.7	2.47	-2.8	2.91	N/A	N/A
21-SM-05	245.7	1.84	-2.7	3.94	N/A	N/A
21-SM-06	717.8	1.84	-3.1	2.35	N/A	N/A
21-SM-07	776.4	1.85	-2.2	3.15	N/A	N/A
21-SM-10	388.7	1.9	2.6	1.78	N/A	N/A
21-SM-11	655.3	1.85	8.7	1.96	N/A	N/A
21-SM-12	800.5	1.87	-2.9	2.16	11	2.11
21-SM-13	561.2	1.87	0.9	0.77	11.6	1.82
21-SM-14	9.5	1.74	9.2	1.96	22.1	1.88
21-SM-15	237.4	1.88	25.9	1.82	39.9	1.85
21-SM-16	210.5	1.86	7	1.71	14.8	1.88
21-SM-17	286.5	1.87	2.2	2.13	60.1	1.99
21-SM-18	155.8	1.87	170.3	2.03	4.2	1.83
21-SM-19	465.7	1.87	-1.4	4.32	104.2	2.01
21-SM-20	761.4	1.86	64.6	1.99	45.5	0.68
22-SM-21	350.9	1.86	7.5	1.82	46.5	0.68
22-SM-22	464.5	1.87	1.3	0.94	21.3	0.59
22-SM-24	203.6	1.85	4.6	1.59	19.8	1.88
22-SM-27	38.9	1.82	0.3	0.31	9	1.68
22-SM-29	1005.9	1.85	3.6	1.98	26.5	1.87
22-SM-30	671.1	1.83	25.3	1.95	96.9	1.97
22-SM-33	631.3	1.8	-0.4	1.18	213.7	2.01
22-SM-34	602.5	1.84	1.2	1.05	62.8	1.91
22-SM-35	247.3	1.87	6.7	1.45	21.1	1.9
22-SM-36	428.8	1.87	5.4	1.8	45.5	1.94
22-SM-37	448	1.86	6.1	1.6	79.5	1.95
22-SM-39	622.3	1.86	5.6	1.76	26.1	1.85
22-SM-40	205.6	1.88	12.9	1.84	103	1.87

^a^ NanoDrop measurements were not performed on the first eight blood clot extractions with the in-house method

All thirty animals were positive for PCR targeting host *cytB* and were therefore suitable for use in *Bartonella* detection. For *gltA* qPCR, nine out of thirty (9/30, 30.0%) spleen samples were positive with Ct values ranging from 24.91 to 35.26 (median 32.47, mean 31.85; Table 2). Eleven out of thirty (11/30, 36.7%) BC samples from in-house extractions were positive on *gltA* qPCR, with Ct values ranging from 21.58 to 35.24 (median 27.86, mean 28.52; Table 2). All nine animals that were positive on spleen testing were also positive on BC testing; two other animals were positive on BC only. Note that three blood samples had not been centrifuged due to low sample volume (Table 2). Four randomly selected *gltA* qPCR + samples sent for Sanger sequencing confirmed the amplified products to be *Bartonella gltA.*

**Table 2:** Results of Bartonella spp. detection via qPCR targeting the gltA gene

Sample ID	Spleen		Blood clot	
	*gltA* qPCR	Ct value	*gltA* qPCR	Ct value
21-SM-02	ND ^a^	-	ND	-
21-SM-03	ND	-	ND	-
21-SM-04	ND	-	ND	-
21-SM-05	ND	-	ND	-
21-SM-06	ND	-	ND	-
21-SM-07	ND	-	ND	-
21-SM-10	positive	33.67 ^b^	positive	27.41
21-SM-11	ND	-	ND	-
21-SM-12	ND	-	ND	-
21-SM-13	ND	-	ND	-
21-SM-14	ND	-	ND	-
21-SM-15	ND	-	ND	-
21-SM-16	ND	-	ND	-
21-SM-17	ND	-	ND	-
21-SM-18	positive	24.91	positive	21.58
21-SM-19 ^c^	positive	35.26	positive	32.57
21-SM-20	ND	-	ND	-
22-SM-21	positive	32.07	positive	35.24
22-SM-22 ^c^	ND	-	positive	33.48
22-SM-24	positive	27.41	positive	28.72
22-SM-27 ^c^	ND	-	ND	-
22-SM-29	ND	-	positive	28.29
22-SM-30	positive	34.90	positive	27.22
22-SM-33	positive	31.48	positive	23.98
22-SM-34	ND	-	ND	-
22-SM-35	ND	-	ND	-
22-SM-36	positive	34.46	positive	27.34
22-SM-37	positive	32.47	positive	27.86
22-SM-39	ND	-	ND	-
22-SM-40	ND	-	ND	-
PC ^d^	positive	24.38	positive	24.38
NTC	ND	-	ND	-
EC	ND	-	ND	-

^a^ ND = not detected by *gltA* qPCR

^b^ Ct values for positive samples are averages of all positive replicates

^c^ clotted whole blood due to low sample volume (i.e. not centrifuged)

^d^ PC = positive control; NTC = no template control; EC = extraction control

## Discussion

Sampling of wild small mammals for *Bartonella* surveillance purposes and disease ecology research has typically relied on collection of whole blood and blood-rich tissues such as spleen and heart [3]. We were able to directly detect *Bartonella* via qPCR from both clotted blood and spleen samples; however, we detected two additional positive animals from the BC samples. Our work builds on previous findings from Schulte Fischedick et al. [11] which compared PCR of tissues with culture of BCs and detected more positives with the latter. We found that direct PCR detection of *Bartonella* from BCs could represent a more accessible and sensitive detection method compared to use of tissues, circumventing the need for culture. BCs may be a better sample type for *Bartonella* detection due to lower concentration of PCR inhibitors in blood samples versus tissues, as well as possible concentration of *Bartonella* in BCs via centrifugation.

Culture of *Bartonella* is laborious, time-consuming and has low sensitivity; similar colony morphologies, minute differences in temperature requirements, and overall differences in cultivability make detecting the presence of co-infection with multiple *Bartonella* spp. via culture difficult [3, 5, 12, 13]. These limitations are important to consider when investigating the ecology of different *Bartonella* spp. in wildlife reservoirs. Few wildlife studies have attempted direct detection via molecular techniques in clotted blood, all of which have utilized kit-based DNA extraction [11, 14–18]. None of the abovementioned studies have reported measurements of DNA extraction quality, and differences in PCR protocols make comparison of PCR results inappropriate. Based on our results, some kit-based DNA extraction methods may be unsuitable for BCs, perhaps due to lack of mechanical disruption of the fibrin mesh [19]. While some kits designed for tissue samples include mechanical disruption steps such as bead tube vortexing, kit-based methods are nevertheless expensive with costs of >$5 USD per sample [19]. Additionally, using tissue samples such as spleen for *Bartonella* spp. investigation requires lethal sampling and although the small mammals used in our study were euthanized for other purposes, methods exist to collect non-lethal blood samples in volumes usable for the described in-house methodology. The use of non-lethal blood samples for *Bartonella* spp. as a viable alternative to tissues reduces invasiveness of sampling and allows for longitudinal studies.

We have demonstrated the successful use of clotted, pelleted blood as a viable sample type for efficient DNA extraction using an in-house protocol, and direct detection of *Bartonella* spp. via real-time PCR. After centrifugation of whole blood and serum separation, pelleted blood cells and clots are typically regarded as a waste product and discarded. We propose that this byproduct of centrifugation may be suitable for hemoparasite molecular testing, particularly hemoparasites of intracellular natures. Therefore, collection of serum from whole blood samples is still possible for use in other testing methodologies, allowing studies to conduct both molecular and serological testing from non-lethal blood samples alone. This is particularly relevant for small mammals like mice, voles, and shrews where blood sample volumes are limited by body mass. Overall, resources and time can be saved through bypassing culture methods and instead conducting DNA extraction and PCR on BCs directly. This practice bears relevance for future *Bartonella* or other hemoparasite disease ecology and surveillance studies, especially those investigating wild small mammal populations.

## Limitations

The sample size used for this study was limited and insufficient to conduct meaningful statistical analysis for comparison of *Bartonella* qPCR performance in BCs versus spleen samples. Additionally, our extractions included only a single kit-based methodology which may not be representative of the capabilities of all commercially available DNA extraction kits. Lastly, our *Bartonella* qPCR reaction only targeted *gltA*; therefore, we cannot guarantee that the described in-house BC DNA extraction protocol would give similar qPCR results with different *Bartonella* gene targets.

### Electronic supplementary material

Below is the link to the electronic supplementary material.

Supplementary Material 1

## Data Availability

No datasets were generated or analysed during the current study.

## Abbreviations

A260/A280: 260 nm/280 nm absorbance ratio for DNA purity assessment
Ct: cycle threshold value; the number of PCR cycles required for the fluorescence threshold to be met during qPCR
DNA: deoxyribonucleic acid
PCR: polymerase chain reaction
qPCR: real-time (‘quantitative’) polymerase chain reaction
USD: United States dollar
