# Supplementary material for: Using clotted, pelleted blood samples for direct molecular detection of Bartonella spp. in small mammal wildlife surveillance studies
Source: BMC Res Notes. 2024 Jul 2;17:184. doi: 10.1186/s13104-024-06841-5 (PMC11221039; doi:10.1186/s13104-024-06841-5)
Supplement: Supplementary file 1 — Supplementary Material 1 [file 13104_2024_6841_MOESM1_ESM.docx]

| **Supplementary Table 1.** Oligonucleotide primers used to target a fragment of cytochrome oxidase B gene (*cytB*) of host animal species via SYBR Green real-time PCR. Host species included the white-footed mouse (*Peromyscus leucopus*), eastern meadow vole (*Microtus pennsylvanicus*), and short-tailed shrew (*Blarina brevicauda*). | | | |
| --- | --- | --- | --- |
| Common name (species) | Forward primer name, sequence | Reverse primer name, sequence | Product size (bp) |
| White-footed mouse (*Peromyscus leucopus*) | JH0885  5’- TAA CTG CCA TGC ATT TGT -3’ | JH0886  5’- CCT GCG GCT AGA ACT GGT AG -3’ | 442 |
| Eastern meadow vole (*Microtus pennsylvanicus*) | JH0883  5’- TAG TAG AAG CTG GGG CAG GA -3’ | JH0884  5’- GAT CCC TGC AGC TAG GAC CAG -3’ | 269 |
| Short-tailed shrew (*Blarina brevicauda*) | JH0881  5’- AGG TAC CGC CCT AAG CAT TT -3’ | JH0882  5’- TCC TGC TAG GTG AAG GGA GA -3’ | 376 |
